# 20 Hz beta stimulation of the subthalamic nucleus improves response inhibition in Parkinson’s disease

**DOI:** 10.1093/braincomms/fcaf474

**Published:** 2025-12-03

**Authors:** Julius Kricheldorff, Tilo Sauer, Karsten Witt

**Affiliations:** Department of Neurology, School of Medicine and Health Science, Carl von Ossietzky University of Oldenburg, 26046 Oldenburg, Germany; Department of Neurology, School of Medicine and Health Science, Carl von Ossietzky University of Oldenburg, 26046 Oldenburg, Germany; Department of Neurology, School of Medicine and Health Science, Carl von Ossietzky University of Oldenburg, 26046 Oldenburg, Germany; Research Center of Neurosensory Science, Carl von Ossietzky University of Oldenburg, 26129 Oldenburg, Germany

**Keywords:** Parkinson’s disease, deep brain stimulation, subthalamic nucleus, inhibitory control, low-frequency stimulation

## Abstract

High-frequency deep brain stimulation of the subthalamic nucleus is used to treat motor symptoms in patients with Parkinson’s disease. There is evidence that low-frequency stimulation in the range of 4–10 Hz may improve cognitive functions. This study investigates whether low-frequency deep brain stimulation of the subthalamic nucleus in the beta band (20 Hz) frequency can improve response inhibition in patients with Parkinson’s disease. In a double-blind crossover design, *N* = 17 participants with Parkinson’s disease performed four neuropsychological experiments, while on their usual dopaminergic medication, under no, standard high-frequency and 20 Hz beta low-frequency deep brain stimulation. The experiments consisted of a response selection task (response execution), a flanker task (conflict monitoring), a Go-NoGo task (automatic inhibition) and a stop-change task (controlled inhibition). Reaction time and response accuracy were analysed using Bayesian mixed-effect models. Participants responded [*m* = 33.6 ms, Bayes factor (BF) = 129.6] slower and [*m* = 6.0%, BF > 1000] more accurately under low-frequency than high-frequency stimulation but not under no stimulation during the response selection task. In the flanker task, participants responded slower under low-frequency than high-frequency stimulation [*m* = 77.0 ms, BF > 1000] but not under no stimulation [*m* = 18.3 ms, BF = 0.1]. We found no performance differences by the stimulation condition of the congruency effect. In the Go-NoGo task, we found low-frequency stimulation slowed responses on uncertain Go trials compared to no stimulation [*m* = 136.8 ms, BF = 30.3] and high-frequency stimulation [*m* = 105.2 ms, BF = 2.5]. Additionally, participants committed fewer errors under low-frequency stimulation than under no and high-frequency stimulation, suggesting that 20 Hz subthalamic nucleus stimulation may improve automatic inhibition. Lastly, in the stop-change task, we found no performance modulation by low-frequency stimulation compared to no and high-frequency stimulation. Our results show that low-frequency beta stimulation may improve aspects of automatic response inhibition in patients with Parkinson’s disease.

## Teaser

In a double-blind crossover study in patients with Parkinson’s disease, Kricheldorff *et al*. found that, compared to OFF and high-frequency stimulation, 20 Hz beta deep brain stimulation improves automatic inhibition but does not affect controlled inhibition.

## Graphical Abstract

**Graphical Abstract:**
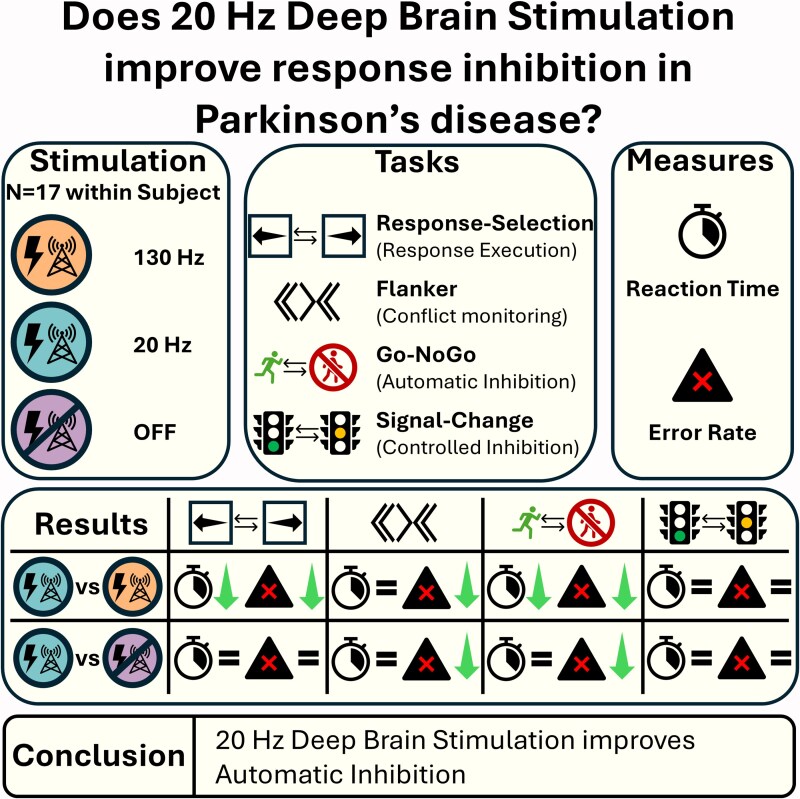

## Introduction

Parkinson’s disease is a progressive neurodegenerative disorder that can only be treated symptomatically.^1^ Motor symptoms in Parkinson’s disease are commonly treated with pharmacological treatments or invasive deep brain stimulation (DBS) at later disease stages. However, the disease burden of Parkinson’s disease depends not only on motor symptoms but also, to a large extent, on non-motor symptoms (e.g. impairments in cognition or dementia).^2^ Currently, effective treatments for non-motor symptoms, such as cognitive deficits, are limited to cognitive^3^ and physical training^4,5^ in mild cognitive impairment and rivastigmine,^6^ an acetylcholinesterase inhibitor in Parkinson’s disease dementia. Thus, it is paramount to investigate new and existing treatments for their potential to improve non-motor impairment.

An effective treatment for motor symptoms of Parkinson’s disease, particularly at later stages, is DBS. This involves inserting electrodes into structures of the basal ganglia, most commonly the globus pallidus interna or subthalamic nucleus (STN), and applying high-frequency electrical current to treat motor symptoms such as tremor, rigidity or bradykinesia. The therapeutic mechanism by which high-frequency DBS of basal ganglia structures alleviates motor symptoms is still a topic of investigation.^7-9^ Basal ganglia structures are not only involved in producing motor output but also play a cardinal role in cognition.^10^ For example, theta band activity in the STN is essential for cognitive control by modulating decision thresholds to prevent premature responding.^11,12^

There have been reports that, depending on stimulation frequency, DBS can be used to attenuate cognitive abilities. High-frequency DBS is often associated with impairments in cognitive abilities, such as inhibition,^13,14^ whereas DBS at low frequencies, defined here as stimulation between 4 and 30 Hz, has been shown to improve cognitive performance.^15,16^ Low-frequency DBS in the delta-theta band range (4–8 Hz) has been reported to improve cognitive control. For example, 5 Hz stimulation of the ventral STN improved accuracy in processing information containing conflict relative to high-frequency DBS or when the stimulator is turned off (OFF DBS),^17^ and 6 Hz STN DBS has enhanced working memory in a Sternberg task.^18^ Moreover, 4 Hz low-frequency DBS of the dorsolateral STN improves interval timing performance.^19^ Verbal^15^ and episodic fluency^16,20^ proactive stopping improves under 10 Hz stimulation, with better efficacy when the contact is located in the dorsal portion of the STN.^20^ Meanwhile, 10 Hz stimulation of the ventral STN may modulate emotional processing.^21^

Cognitive control encompasses a set of executive functions that enable flexible, goal-directed behaviour by coordinating mental processes to override automatic responses when they conflict with current intentions.^22^ These executive functions include selective attention, conflict monitoring^23,24^ and response inhibition. Within this framework, response inhibition represents a critical component that allows organisms to suppress inappropriate motor responses, whether these responses are already initiated or prepared for execution.^25,26^ The STN plays a cardinal role in this inhibitory control system through its position in the hyperdirect pathway, which provides rapid cortical input that can modulate motor output before responses become overt.^11,27,28^

The term inhibition can differ in meaning depending on the discipline.^25^ When we speak of inhibition in this context, we refer to motor inhibition—the stopping or delay of an overt response.^25,26^ Laboratory paradigms for studying motor inhibition include the Go-NoGo task or the stop-signal task, where participants are required to inhibit an overt response.^25^ Both tasks differ in what aspects of inhibition they assess. Stopping requires prefrontal regions, in particular the right inferior frontal gyrus, the pre-supplementary motor area and the basal ganglia, in particular the STN.^28,29^ Response inhibition can be achieved through both automatic and controlled mechanisms, depending on the consistency of stimulus-stop associations.^30^ Verbruggen and Logan^30^ demonstrated that when stimuli are consistently mapped onto stopping (as typically occurs in Go-NoGo tasks), automatic response inhibition develops through learned stimulus-stop associations that can be retrieved from memory. In contrast, when stimulus-stop mappings are inconsistent (as in standard stop-signal tasks), inhibition relies primarily on controlled, top-down processes. Thus, the Go-NoGo and stop-signal paradigms may not be equivalent measures of inhibitory control, as they differentially engage automatic versus controlled inhibitory processes.^30^

The STN also plays a cardinal role in cognitive control, response inhibition and motor impulsivity^31,32^ via the hyperdirect pathway.^19,27^ In particular, modulation of STN beta oscillations is associated with controlled and reactive movement inhibition.^33,34^ For example, Kühn *et al*.^33^ found the degree of beta band desynchronization in the STN to be associated with response execution and response inhibition, with more desynchronization associated with movement execution and less with movement inhibition in a Go-NoGo task. The observed average differences in beta could reflect modulation in beta burst dynamics, not sustained beta changes.^35,36^ Increased beta burst rates in the STN predict stopping in the stop-signal task,^35^ which precedes and predicts beta burst activity in the sensorimotor cortex—known to be associated with inhibitory control (see Diesburg *et al*.^35^). Beta band amplitude has further been associated not only with successful stopping but also with global corticospinal excitability.^37^

Given the importance of STN activity in the beta frequency range for response execution and response inhibition, we tested therapeutic high-frequency stimulation (HFS), no stimulation and beta frequency stimulation (20 Hz). To evaluate possible effects on cognitive and motor functions, we employed a hierarchical test battery examining response execution, cognitive control and reactive and controlled inhibition in patients with Parkinson’s disease. To this end, we used a simple response selection task (choice reaction time task), a conflict monitoring task with interference (flanker task), and two tasks involving controlled inhibition (stop-change task) and reactive inhibition (Go-NoGo task).^30,38,39^ Participants were tested three times: OFF DBS, their usual ≥130 Hz DBS or beta 20 Hz DBS. We hypothesized that beta frequency stimulation would improve controlled and automatic inhibition but, unlike 10 Hz stimulation, should not affect performance in the response selection or cognitive control task.

## Materials and methods

### Study design and participants

The study was approved by the medical ethics committee of the Christian-Albrechts University of Kiel (A134/00) in accordance with the Declaration of Helsinki.^40^ We recruited 19 participants with Parkinson’s disease who had undergone DBS surgery at the Department of Neurology of the University Hospital Schleswig-Holstein. The sample size was determined by how many participants we could feasibly enrol within a year at our institution. We excluded one participant because they could not understand the task instructions. One patient discontinued the study due to transient side effects. The final sample consisted of 17 participants (5 female). All participants diagnosed with Parkinson’s disease fulfilled the International Parkinson and Movement Disorder Society criteria for Parkinson’s disease^41^ and the following inclusion criteria: (i) bilateral STN DBS for at least 3 months with the Medtronic 3389 DBS electrode, (ii) a Mini-Mental State Examination (MMSE) ≥ 25, (iii) no additional psychiatric diseases and (iv) visual acuity (with or without corrective lenses) of >0.9 and intact colour vision. Further, we recorded levodopa equivalent daily dose (LEDD),^42^ disease subtype, the most affected side, Hoehn and Yahr scale rating, disease duration and time since DBS implantation (see Table 1).

**Table 1:** Baseline characteristics

Age (in years)	63.6 (7.1)
Disease duration (in years)	15.1 (6.1)
DBS treatment duration (in years)	3.2 (2.3)
MMSE	28.2 (1.6)
Hoehn and Yahr stage (1–5)	2.8 (0.5)
LEDD (in mg)	524.4 (398.6)
Most affected side	
Left	*N* = 6
Right	*N* = 11
Disease subtype	
Akinetic-rigid	*N* = 9
Tremor-dominant	*N* = 3
Equivalent type	*N* = 5

Displays patients’ baseline characteristics, including demographics, stage of the disease and cognitive, affective, motor and drug information. Clinical characteristics of the patient group are displayed as means, with standard deviations shown in parentheses or counts.

### Stimulation settings

**Figure 1:**
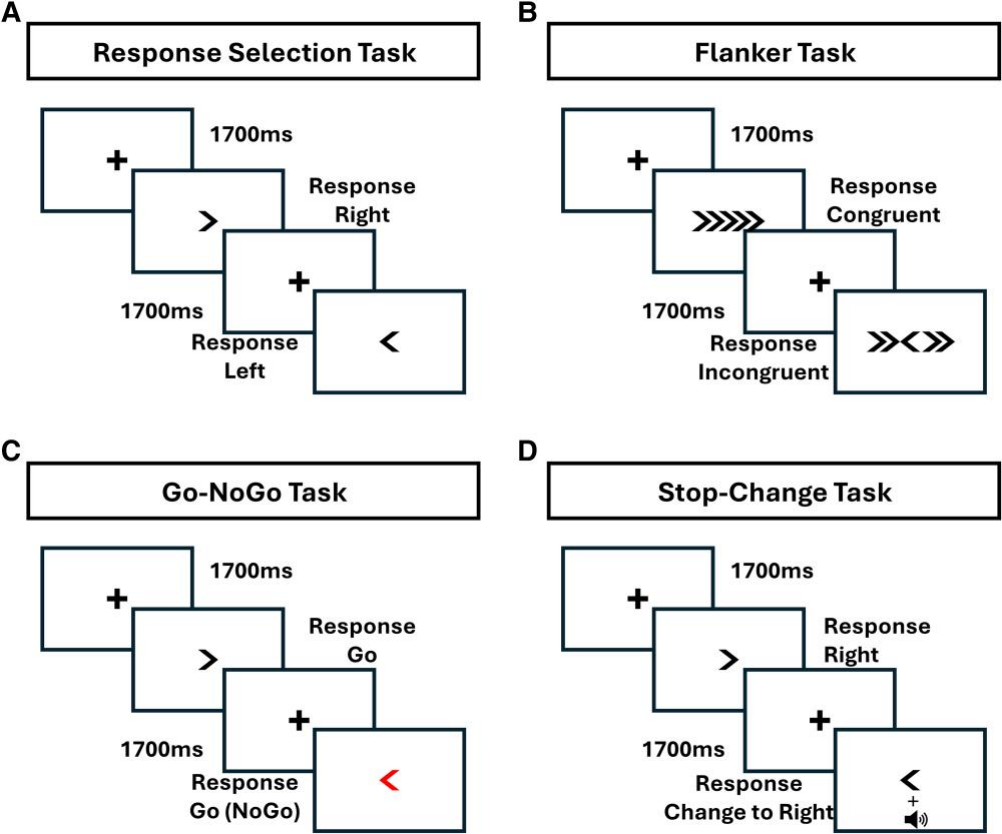
**Task description.** Experimental tasks performed in each stimulation session. (**A**) Task diagram of the response selection task. A fixation was followed by the target stimulus of an arrow indicating the correct response direction until a response was indicated. (**B**) Task diagram of the flanker task. A fixation was followed by the target stimulus in the centre of the screen, flanked by arrows that could point either in the same direction as the target or in the opposite direction (incongruent trials). The flanked target arrow remained on the screen until a response was indicated. (**C**) Task diagram of the Go-NoGo task. A fixation was followed by the target stimulus of an arrow indicating the correct response direction, either in black or in red. Black arrows were always Go responses (certain Go trials). In contrast, a specific direction for red arrows corresponded to withholding a response (NoGo trials) and the other direction to Go responses (uncertain Go trials). The target stimulus was displayed until a response was indicated during Go trials or no response was indicated for 2000 ms during NoGo trials. (**D**) Task diagram of the stop-change task. A fixation was followed by the target stimulus of an arrow indicating the correct response direction until a response was indicated. During change trials, the display of the target stimulus was followed by a tone after the ‘CSD’ time, prompting participants to respond opposite to the direction displayed.

Participants performed the same set of tasks (see Fig. 1) under three different bilateral stimulation conditions with a 20 min washout period: no stimulation (OFF), their standard (≥130 Hz) clinical high-frequency DBS and 20 Hz beta low-frequency stimulation (LFS). For the LFS, we used the same pulse width and amplitudes as for the clinically effective ≥130 Hz stimulation (see Table 2). To mitigate potential order effects, the presentation order of the three stimulation conditions was permuted and counterbalanced across participants using a Latin square design, with participants randomly assigned to one of the six possible permutations. The order in which the tasks were presented was fixed. Participants were presented first with a response selection task, followed by a flanker task, a Go-NoGo task, a stop-change task and an MDS-Unified Parkinson’s Disease Rating Scale (UPDRS-III) motor examination assessment. The experiment lasted up to 3 h, with each experimental stimulation session lasting 20–30 min on average. Participants remained on their regular dopaminergic medication regimen throughout the experimental procedures. All experimental sessions were double-blind, with an additional experimenter performing the DBS programming before each session.

**Table 2:** Participant demographics and DBS settings

ID	Age	Gender	MMSE	Contact		Amplitude		Pulse width		Frequency		UPDRS-III		
				Left	Right	Left	Right	Left	Right	Left	Right	OFF	LFS	HFS
1	52	M	27	9.1	1	3.9	1	60	60	180	180	77	73	32
2	54	F	28	5.6	1.3	4	3.3	60	60	180	180	33	25	16
3	65	F	29	5.7	1.3	2.3	2.45	60	60	160	160	35	46	22
4	62	M	29	5	1	4.2	4.2	60	60	180	180	23	33	26
5	63	M	26	8.9	1.2	2.5	3	60	60	160	160	31	31	19
6	69	M	30	5.7	2	3	3.5	60	60	180	180	18	10	10
7	66	M	29	6	2	2.7	2.3	60	60	180	180	27	26	16
8	60	M	29	9	1	1.4	1.4	60	60	130	130	48	45	31
9	66	M	27	9	1	3.2	2.8	60	60	160	160	48	34	41
10	76	F	25	10	2	2.4	4	60	60	130	130	45	28	31
11	68	F	28	6	2	2.1	2.2	60	60	130	130	29	34	21
12	69	M	26	5	2.3	2	3	60	60	180	180	64	51	34
13	52	M	30	9.1	1	3	1	60	60	130	130	23	26	18
14	75	F	30	5.6	1.3	3.9	1.8	60	60	180	180	17	17	14
15	62	M	28	5	1	2.2	3.5	60	60	150	150	22	22	19
16	65	M	29	6	2	2.7	4	60	60	210	210	31	31	31
17	57	M	30	5.6	1	3.5	3.2	60	60	180	180	36	36	28

### Experimental paradigms

All tasks were programmed using Visual Basic 6.0. During each task, participants were seated ∼50 cm away from a 15-inch monitor. They indicated their responses via two button boxes marked with a left-arrow symbol for a left-hand response and a right-arrow symbol for a right-hand response. Ten practice trials preceded each task. For each task, response time (RT) and accuracy were recorded. Symbols displayed in the tasks were either black or red on a white background (see Fig. 1). For the response selection task, a fixation after 1700 ms was followed by an arrow symbol indicating the required response. Participants performed a total of 80 trials in one session. In the flanker task, participants were required to indicate the direction of a target arrow presented by two irrelevant arrows left and right that point in the same (congruent: >>**>**>> or <<**<**<<) or opposing directions (incongruent: >>**<**>> or <<**>**<<). The task consisted of 100 trials, with half of the trials being congruent and the other half incongruent. In the stop-change task, participants saw an arrow on the screen in the direction in which they had to respond. In 30 out of 120 trials, the display was accompanied by a tone, indicating that participants had to switch their responses. For example, if a left arrow was displayed, participants had to respond by pressing the right response button. The tone was delayed to the display presentation (initially set at 50 ms) and was adjusted using a staircase tracking procedure to determine the individual participant’s ‘change signal delay’ (CSD) time. Every time participants successfully stopped (responded correctly), the CSD time was increased by 50 ms, and every time the participants failed to respond to the stop signal (responded incorrectly), the CSD was decreased by 50 ms. For the Go-NoGo task, participants performed 80 trials in total. Half of the trials consisted of black arrows, the other half red arrows. Black arrows always corresponded to Go responses (certain Go trials), and participants had to respond in the direction the arrow indicated. For arrows in red, one direction would correspond to a Go response (uncertain Go trials), and the other would correspond to withholding a response (NoGo trials). For half the participants, an arrow pointing to the left indicated withholding a response (in total, 20 NoGo trials), and an arrow pointing to the right would indicate that Go responses (in total, 20 uncertain Go trials) and vice versa reversed for the other half of the participants. Thus, there were 40 certain Go trials in black, 20 uncertain Go trials in red and 20 NoGo trials. NoGo trials lasted up to 2 s or until a response was indicated. For the response selection, flanker and stop-change tasks, stimuli remained visible on the screen until participants recorded their responses. Each trial was preceded by a fixation cross lasting 1700 ms for all tasks.

### Statistical analysis

All analyses were performed in R (R-4.1.3),^43^ and data were visualized with the ggplot2^44^ and ggdist^45^ packages.

To analyse the UPDRS-III and signal-change RT, we calculated the difference score between stimulation conditions and used a one-sample *t*-test implemented in the BayesFactor^46^ package. We analysed single trial data for all other tasks and used Bayesian mixed-effect models for the statistical analyses, which were implemented using the brms package.^47^ For the RT analysis, we excluded correctly answered trials <200 ms or >3000 ms (2.8% of the Go-NoGo task data, 2.1% of the flanker task data, 0.3% of the response selection task data and 3.7% of the stop-change task data). Go-NoGo, flanker and response selection task RT data were analysed using a shifted log-normal likelihood function to account for the right skew of the RT distribution. Moreover, the error data of the three tasks were analysed using logistic regression (Bernoulli likelihood with a logit-link function). We used dummy-coded variables to calculate the main effects of stimulation conditions (LFS, high-frequency DBS, and OFF) in the stop-change task. For the analysis of the flanker task, we included another dummy-coded main effect for the experimental conditions (congruent versus incongruent) and the interactions with the stimulation condition. The error data of the Go-NoGo task were similarly analysed with a dummy-coded variable for the stimulation condition and trial type (certain Go, uncertain Go, and NoGo) and their interaction. To analyse the Go-NoGo RT data to quantify slowing on uncertain Go trials (the target was red), we calculated the difference between certain Go and uncertain Go by the stimulation condition and their interaction. Moreover, for all analyses, we included a random intercept for participants to account for individual-specific random variability. We evaluated parameter estimates in per cent or milliseconds by calculating the estimated marginal mean effects from the posterior distributions for both the error and RT data. For the stop-change task, we computed the signal-change reaction time (equivalent to stop-signal reaction time^48^) using the integration method^49,50^ for each participant and stimulation condition. Differences in signal-change time between stimulation conditions were then calculated via *t*-tests using the aforementioned BayesFactor^46^ package. Model definitions and prior distributions can be found in the supplement (see Supplementary Equations 1–6).

We used bridge sampling^51^ to calculate Bayes factors (BFs) to quantify evidence for or against including a particular parameter. Here, the BF can be understood as the likelihood of the alternative model containing the parameter of interest over the likelihood of the null model with the parameter of interest set to a constant 0. A BF < 1 is evidence in favour of the null model excluding the parameter, and a BF > 1 is evidence in favour of including the parameter. We evaluated the evidential strength with the criteria for BFs defined by Jeffreys^52^: (i) 1 < BF < 3 (for the null model: 1/3 < BF < 1) is anecdotal evidence for the alternative model, (ii) 3 < BF < 10 (for the null model: 1/10 < BF < 1/3) is moderate evidence for the alternative model, (iii) 10 < BF < 30 (for the null model: 1/30 < BF < 1/10) is strong evidence for the alternative model, and (iv) BF > 30 (for the null model: BF < 1/30) is substantial evidence for the alternative model.

We used four chains for the Markov chain Monte Carlo sampling. The first 2000 iterations of each chain were discarded as warm-ups, and the following 10 000 iterations were used to sample from the posterior distribution. Model convergence was verified by visually examining the chain trace plots and confirming that the scale reduction factor *R* for all parameters was close to 1 and >1.1. We assessed the model fit by comparing the agreement of the estimates of the posterior predictive distribution with the observed data (see Supplementary Figs 1–6).

## Results

### Baseline characteristics

Baseline characteristics of the participants are given in Table 1.

### Behavioural results

#### UPDRS-III

We found substantial evidence for a reduction in UPDRS-III for high-frequency DBS relative to OFF status [*m* = −11.7, 95% credible interval (CI) = (−16.6, −5.7), *t*(16) = −4.2, *P* < 0.001, BF = 50.3] and strong evidence for a reduction of high-frequency DBS relative to low-frequency DBS [*m* = −9.4, 95% CI = (−15.1, −3.6), *t*(16) = −3.5, *P* = 0.003, BF = 13.6]. Moreover, we found anecdotal evidence in favour of no difference for low-frequency DBS relative to OFF status [*m* = −2.3, 95% CI = (−6.3, 1.7), *t*(16) = −1.2, *P* = 0.24, BF = 0.48].

#### Response selection task results

**Figure 2:**
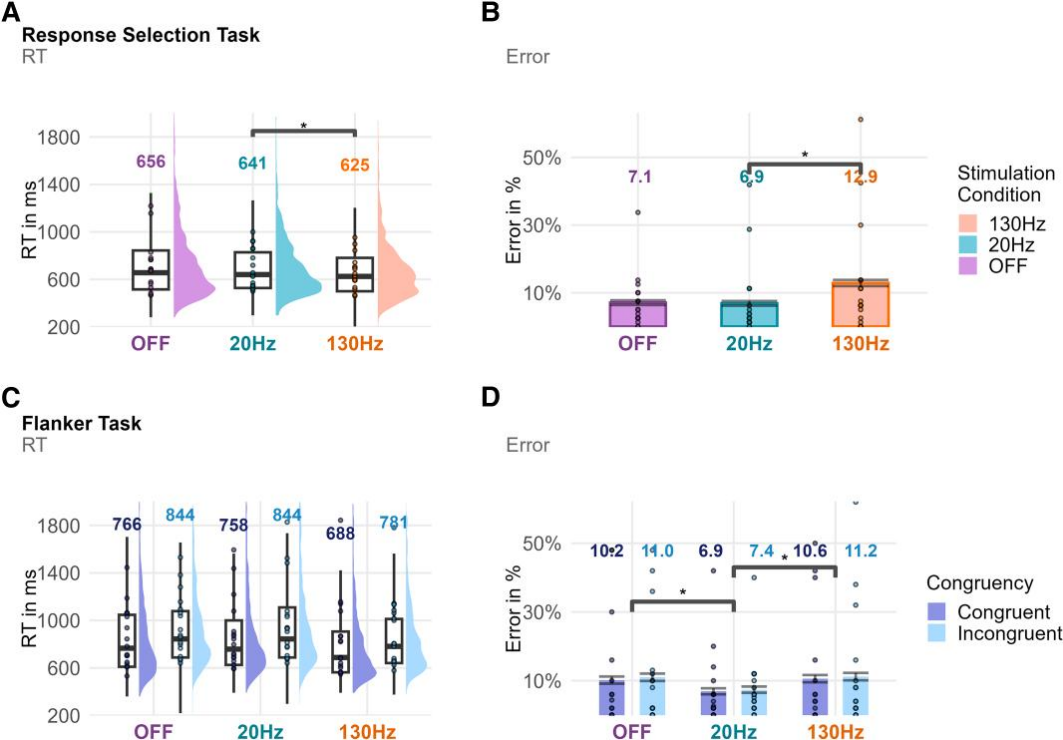
**Results from the response selection task and flanker task.** Display of reaction time and error data (*N* = 17) by the stimulation condition for the response selection task (**A, B**) and the flanker task (**C, D**). Numbers above the reaction time distributions (**A, C**) correspond to the median reaction time, and numbers above the bar graphs of the error data (**B, D**) correspond to the mean error. Differences between conditions that exceed a BF threshold of 3, as assessed by Bayesian mixed-effect models, are marked with an *. There is substantial evidence (BF = 129.6) that high-frequency DBS produces faster responses than low-frequency DBS in the response selection task. For response selection task error rates, there is substantial evidence (BF > 1000) that low-frequency DBS reduces errors compared to high-frequency DBS. In the flanker task, there is substantial evidence (BF > 1000) that low-frequency DBS produces slower responses than high-frequency DBS. For flanker task errors, there is substantial evidence that low-frequency DBS reduces errors compared to both high-frequency DBS (BF = 371.8) and OFF stimulation (BF = 161.1).

We found that participants responded faster under high-frequency DBS than under low-frequency DBS [*m* = −33.6 ms, 95% CI = (−50.2 ms, −16.7 ms), BF = 129.6] but not OFF stimulation compared to low-frequency DBS [*m* = 6.5 ms, 95% CI = (−10.9 ms, 23.2 ms), BF = 0.06] in the response selection task (see Fig. 2A). Moreover, the analysis of the error data showed that participants committed fewer errors under beta low-frequency DBS compared to high-frequency DBS [*m* = −6.0%, 95% CI = (−8.1%, −3.9%), BF > 1000] but not compared to OFF stimulation [*m* = 0.001%, 95% CI = (−2.0%, 1.7%), BF = 0.1] (see Fig. 2B).

#### Flanker task results

We found substantial evidence that participants reacted slower under beta low-frequency DBS compared to high-frequency DBS [*m* = 77.0 ms, 95% CI = (58.3 ms, 96.0 ms), BF > 1000] and moderate evidence favouring the null hypothesis of no difference for beta low-frequency and OFF stimulation [*m* = 18.3 ms, 95% CI = (−1.4 ms, 37.4 ms), BF = 0.1] in the flanker task (see Fig. 2C). We observed the typical congruency effect with an increase in reaction time on incongruent trials compared to congruent trials [*m* = 105.3 ms, 95% CI = (90.0 ms, 120.9 ms), BF > 1000] but no evidence for a difference in congruency effects between low-frequency DBS and high-frequency DBS [*m* = −19.9 ms, 95% CI = (−57.6 ms, 16.7 ms), BF = 0.12] or beta low-frequency DBS and OFF stimulation [*m* = 8.0 ms, 95% CI = (−30.7 ms, 46.6 ms), BF = 0.10] (see Supplementary Fig. 8A). In terms of error data, we found substantial evidence that participants committed fewer errors under beta low-frequency DBS compared to high-frequency DBS [*m* = −3.7%, 95% CI = (−5.4%, −1.9%), BF = 371.8] and substantial evidence of fewer errors under beta low-frequency compared to OFF stimulation [*m* = −3.4%, 95% CI = (−5.2%, −1.6%), BF = 161.1] (see Fig. 2D). Concerning conflict monitoring, we found moderate evidence against a congruency effect [*m* = 0.6%, 95% CI = (−0.9%, 2.1%), BF = 0.1] and further no effect of stimulation status on the congruency effect in the error data (see Supplementary Fig. 8B).

#### Go-NoGo task results

**Figure 3:**
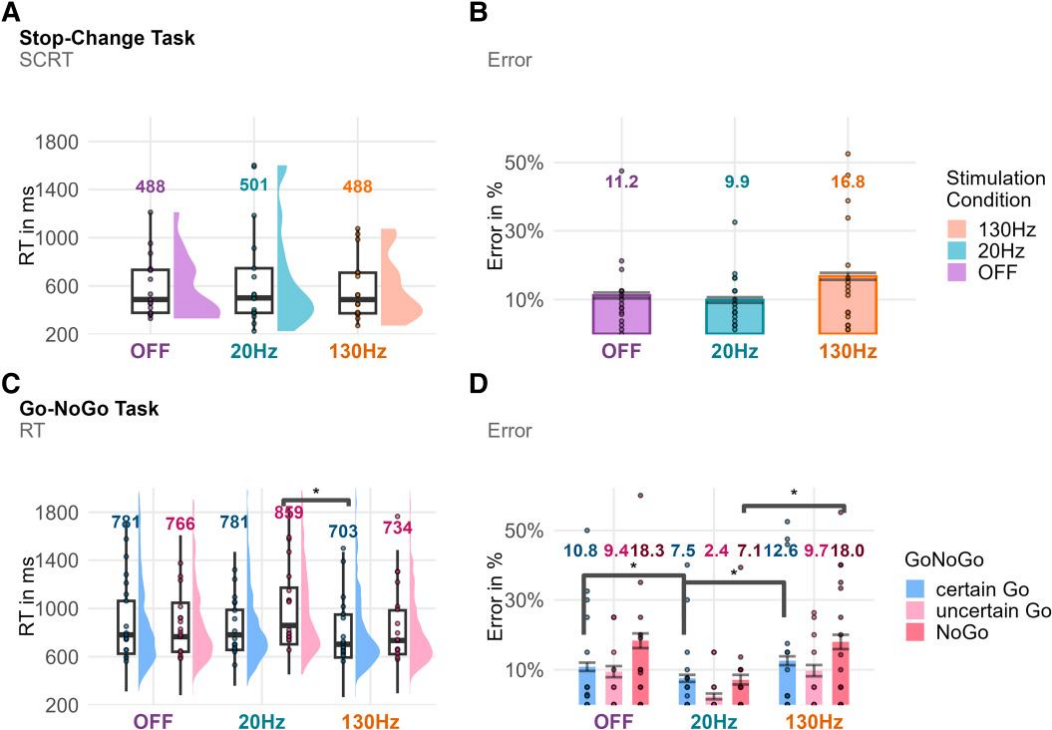
**Results from the stop-change task and Go-NoGo task.** Display of reaction time (SCRT for the stop-change task) and error data (*N* = 17) by the stimulation condition for the stop-change task (**A, B**) and the Go-NoGo task (**C, D**). Numbers displayed above the reaction time distributions (**A, C**) correspond to the median reaction time, and numbers displayed above the bar graphs of the error data (**B, D**) correspond to the mean error. Differences between conditions that exceed a BF threshold of 3, as assessed by Bayesian mixed-effect models, are marked with an *. No differences were found between stimulation conditions in the stop-change task. For the Go-NoGo task, there is strong evidence (BF = 30.3) that low-frequency DBS increases response caution (slower responses on uncertain versus certain Go trials) compared to OFF stimulation and anecdotal evidence (BF = 2.5) for increased caution compared to high-frequency DBS. There is substantial evidence that low-frequency DBS reduces errors compared to high-frequency DBS on uncertain Go trials (BF > 1000) and NoGo trials (BF > 1000) and compared to OFF stimulation on uncertain Go trials (BF = 691.9) and NoGo trials (BF > 1000).

To assess response caution, we compared how Go responses differed when a Go response was certain (black Go trials) versus uncertain Go trials when a NoGo response was equally likely (red Go trials). We found strong evidence that participants under beta low-frequency DBS answered more slowly/cautiously on uncertain Go trials (equal probability of Go and NoGo responses) versus certain Go trials than participants under OFF stimulation effects [*m* = 136.8 ms, 95% CI = (75.6 ms, 198.0 ms), BF = 30.3]. Similarly, we found anecdotal evidence of slowing on uncertain trials under beta low-frequency DBS compared to high-frequency DBS [*m* = 105.2 ms, 95% CI = (46.7 ms, 164.2 ms), BF = 2.5] (see Fig. 3C). A subsequent analysis of the error data revealed a similar pattern. We found substantial evidence that participants committed fewer errors under beta low-frequency DBS compared to high-frequency DBS on uncertain Go trials [*m* = −6.8%, 95% CI = (−10.1%, −3.6%), BF > 1000] and on NoGo trials [*m* = −10.3%, 95% CI = (−14.5%, −6.0%), BF > 1000] (see Fig. 3D). Moreover, we found substantial evidence that participants committed fewer errors under beta low-frequency DBS compared to high-frequency DBS on uncertain Go trials [*m* = −6.5%, 95% CI = (−9.8%, −3.3%), BF = 691.9] and substantial evidence on NoGo trials [*m* = −10.6%, 95% CI = (−15.0%, −6.3%), BF > 1000].

#### Stop-change task results

We found anecdotal evidence for the null hypothesis of no difference in stop-change reaction time (SCRT) between conditions for the comparison of low-frequency DBS and high-frequency DBS [*m* = 114.7 ms, 95% CI = (−88.3 ms, 317.7 ms), *t*(16) = 1.20, *P* = 0.25, BF = 0.46] and low-frequency DBS compared to OFF stimulation [*m* = 75.1 ms, 95% CI = (−96.7 ms, 246.9 ms), *t*(16) = 0.93, *P* = 0.37, BF = 0.36] (see Fig. 3A). Moreover, we found no evidence for differences in Go RTs between stimulation conditions or Go response error rates.

### *Post hoc* analysis Go-NoGo task results

To investigate potential modulatory effects of participants’ dopaminergic and motor status, we examined correlations between medication doses (LEDD scores), motor symptoms (UPDRS-III scores), response slowing on Go trials (reaction time difference between uncertain and certain Go trials) and error rates. We calculated Pearson’s correlation coefficients for each stimulation condition with significance set at *α* = 0.05.

**Figure 4:**
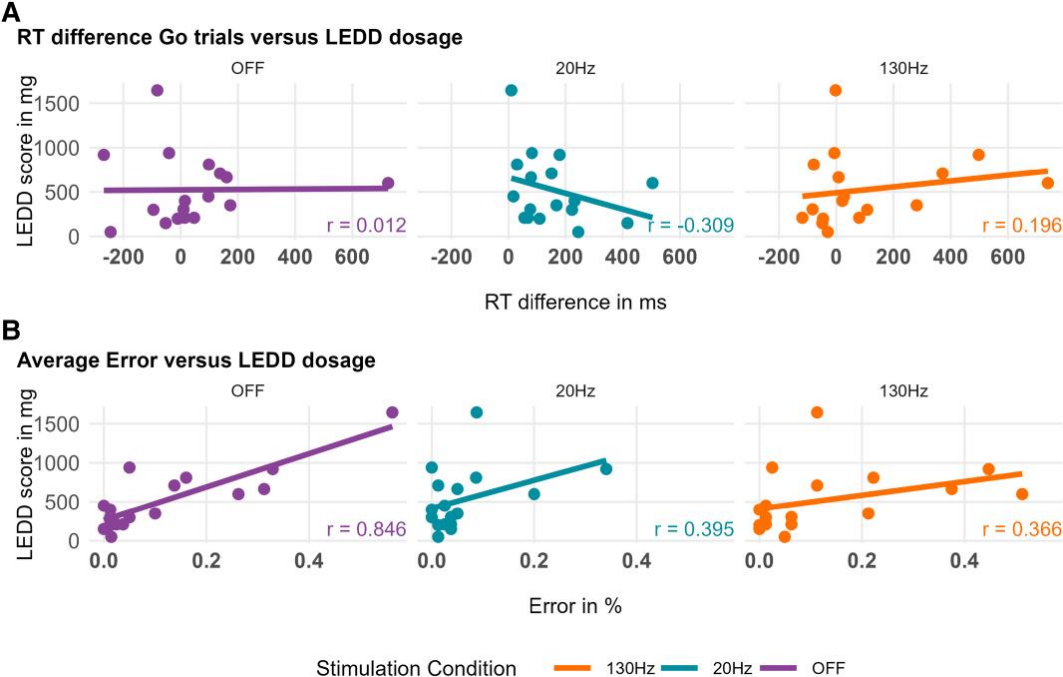
**Results of *post hoc* analyses.** Relationship between dopaminergic medication and task performance across stimulation conditions. (**A**) Correlation between LEDD and response slowing, measured as the reaction time difference between uncertain and certain Go trials. (**B**) Correlation between LEDD and error rates. Pearson’s correlation coefficients (*N* = 17) are shown for each stimulation condition. Solid lines represent linear regression fits. For the *post hoc* Go-NoGo analysis, no significant associations were found between reaction time differences and LEDD scores across any stimulation condition. For error rates, there was a significant, strong positive correlation with LEDD scores in the OFF stimulation condition (*r* = 0.846, *P* < 0.001), but no significant correlations were found between low-frequency DBS or high-frequency DBS error rates and LEDD.

We found no significant associations between reaction time differences and LEDD scores across any stimulation condition (see Fig. 4). For error rates, we observed a strong positive correlation with LEDD scores only in the absence of DBS (*r* = 0.846, *P* < 0.001) but not during low-frequency DBS (*r* = 0.395, *P* = 0.12) or high-frequency DBS (*r* = 0.366, *P* = 0.15). Although not significant during DBS conditions, the direction of this relationship remained consistent across all conditions.

The analysis of the association between UPDRS-III scores and both slowing on Go trials and error rates showed no significant correlation (see Supplementary Fig. 7).

## Discussion

In a randomized, double-blind, crossover design, we investigated the effect of bilateral 20 Hz beta stimulation of the STN on inhibition in patients with Parkinson’s disease. To this end, we performed four experiments assessing simple reactions (response-election task), conflict monitoring (flanker task), automatic inhibition (Go-NoGo task) and controlled inhibition (stop-change task) under high-frequency DBS, beta low-frequency DBS and no stimulation. In line with our hypothesis, we found strong evidence that beta low-frequency DBS improves automatic inhibition, marked by increased accuracy when inhibiting a response and increased slowing on trials where stopping could be expected. The effect of beta low-frequency DBS was specific to automatic inhibition, as we found no effect on simple reactions and conflict monitoring abilities. However, contrary to our expectation, we saw no evidence that beta low-frequency DBS improves controlled inhibition as measured by the stop-change task. We also have to mention the possibility that the stop-change task was not suitable to detect controlled inhibition. For instance, Krämer *et al*.^53^ have previously shown that cortical electrophysiological markers of inhibition differ between the stop-change and stop-signal tasks.

### Beta stimulation effects on automatic and controlled inhibition

We show improved automatic inhibition under bilateral beta low-frequency DBS. Participants made fewer NoGo errors and responded slower on Go trials primed by NoGo. Our results provide further causal evidence for the pivotal role of the STN for inhibition in general and indirect evidence of STN beta oscillations. Beta oscillations in the STN have been reported to be relevant for inhibition for a long time,^33,35^ and specifically, beta burst dynamics predict stopping behaviour.^35,36^ Considering the cardinal role of beta burst dynamics in inhibition, low-frequency DBS may modulate beta burst dynamics in the STN. However, since no local field potentials of the STN were recorded during the experiment, we can only speculate about how beta low-frequency DBS may affect STN activity. In a *post hoc* analysis, we further explored whether the observed effects of LFS may have been modulated by dopaminergic medication or motor status. Motor status showed no association with either response slowing on uncertain Go trials or error rates. Similarly, dopaminergic medication levels did not correlate with response slowing. However, higher dopaminergic medication doses (LEDD) were significantly associated with increased error rates when DBS was turned off but not during high-frequency stimulation or LFS, suggesting enhanced error susceptibility in highly medicated patients without stimulation. Consequently, we do not consider the effect of dopaminergic medication as explanatory for the observed effects of LFS on Go-NoGo task performance for two reasons: first, we found no evidence for medication effects on response slowing, and second, while the relationship was only significant without stimulation, its direction remained consistent across DBS conditions. Our results regarding the effect of beta low-frequency DBS on controlled inhibition as measured using the stop-change task support the null hypothesis of no effect. This may suggest that the observed modulation of beta low-frequency DBS of the STN is limited to automatic and not controlled inhibition. However, we acknowledge the methodological limitations of our task. To assess motor, conflict monitoring and inhibitory domains, we could only perform a short version of the stop-change task to limit the strain on our participants. The number of trials used here was lower than recommended, according to a recent consensus paper on the stop-signal task.^54^ Since a lower trial number may lead to unreliable stop-signal (here signal-change) reaction time estimates, this result warrants further replication.

### Beta low-frequency DBS is specific to inhibition

Our results support growing evidence that the STN modulates cognitive abilities by frequency. In our study, beta low-frequency DBS led to improvements in automatic inhibition but did not affect the congruency effect as a measure of conflict resolution/cognitive control. The lack of an effect of beta low-frequency DBS on conflict monitoring is consistent with evidence linking conflict monitoring and the STN primarily via variation in theta power.^55^ The compartmentalization of cognitive abilities by frequency in the STN has also been shown in recent work by Salehi *et al*.^18^ on the effect of theta STN DBS on working memory performance. Here, improvements in working memory were restricted to theta but not beta or gamma frequency stimulation. We did see an improvement in accuracy in the flanker task paradigm of beta low-frequency DBS compared to no stimulation. However, this improvement cannot be attributed to the classical speed-accuracy trade-off, as it was not accompanied by an increase in RT.

The observed slowing in the Go-NoGo task’s uncertain condition presents an interesting theoretical puzzle. Typically, response slowing under conflict conditions is attributed to the STN’s ‘hold-your-horses’ function,^11^ which is associated with low-frequency oscillations in the 2–8 Hz range^56,57^ rather than beta frequencies. However, our task design may fundamentally differ from classical conflict paradigms. Rather than creating conflict between two competing Go responses, the uncertain Go condition creates conflict between a prepared Go response and a prepared stopping response. This distinction is crucial because it transforms the task from one of conflict resolution to one primarily involving response inhibition mechanisms.

From the perspective of automatic versus controlled inhibition, our results initially appear contradictory. Verbruggen and Logan’s^30^ framework suggests that automatic response inhibition develops through consistent stimulus–response mappings, while the uncertain Go condition involves inconsistent mappings that should primarily engage controlled inhibition. If beta stimulation selectively enhanced only one type of inhibition, we would expect differential effects on certain stop trials versus uncertain Go trials. Instead, we observed improvements in both conditions, suggesting that 20 Hz stimulation may enhance inhibitory control more broadly rather than selectively modulating specific inhibitory mechanisms. This interpretation aligns with beta oscillations’ general role in motor suppression,^33,35^ where enhanced beta activity could strengthen the overall capacity for response inhibition regardless of whether it operates through automatic or controlled pathways. The absence of similar effects in the stop-change task likely reflects methodological limitations rather than theoretical inconsistencies, as discussed in the following section.

### Limitations

In this study, we tried to balance task and intervention durations and resultant data quality. Consequently, it is possible that our stop-change task paradigm may not have had sufficient trials to assess the effect of beta low-frequency DBS on controlled inhibition. Future research should use an experimental paradigm following consensus-based guidelines by Verbruggen *et al*.^54^ to determine whether beta low-frequency DBS improves inhibition in general or is specific to automatic inhibition. Moreover, we need a more mechanistic understanding of how beta low-frequency DBS may influence STN activity to enhance inhibition. To answer this question, further inquiry using additional recording of STN local field potentials during beta low-frequency DBS is necessary.

### Future direction

Beyond its interest in our fundamental understanding of inhibitory control and dysfunction in Parkinson’s disease, these findings should be further tested for their clinical utility. In addition to motor dysfunction, patients with Parkinson’s disease often suffer from executive and inhibitory dysfunctions.^58-60^ Low-frequency DBS may positively affect executive functioning. However, as shown here, as well as previous research,^61,62^ beta and other low-frequency DBS can lead to worse outcomes in motor performance in patients with Parkinson’s disease. While worsening motor status can be prevented by keeping the volume of tissue activated constant,^18^ low-frequency DBS by itself is unlikely to yield clinically meaningful improvements for patients. Low-frequency DBS should be combined with high-frequency DBS to relieve motor symptoms in patients. For example, if used in the context of adaptive DBS with other frequencies at different contacts, DBS might become a tool to improve both cognitive and motor symptoms in patients with Parkinson’s disease.^18,20,63^ The feasibility of such an approach has been shown in a recent study by Jia *et al*.^64^ Here, high-frequency DBS was successfully combined with mid-frequency DBS (60–80 Hz) to improve both UPDRS-III scores and symptoms of freezing of gait. Lastly, aside from stimulation frequency, lead trajectory and electrode position are known to be associated with neuropsychiatric outcomes of high-frequency DBS.^31,65^ Electrode locations were determined independently of this study based on clinical criteria to ensure the effectiveness of high-frequency DBS. It would be interesting to see to what degree the effect of beta low-frequency DBS depends on the STN’s somatosensory and cognitive zones.^31,66,67^

## Conclusion

We show that beta low-frequency DBS can modulate stopping in patients with Parkinson’s disease. In conjunction with other low-frequency DBS protocols,^16-18,20^ our results provide a promising starting point for new treatment avenues for non-motor symptoms.

## Supplementary Material

fcaf474_Supplementary_Data

## Data Availability

Because the information contained could compromise the privacy of research participants and the lack of consent, the data cannot be made publicly available. The code used for the analysis can be found at https://osf.io/s4utw/.
