# Supplementary material for: 20 Hz beta stimulation of the subthalamic nucleus improves response inhibition in Parkinson’s disease
Source: Brain Commun. 2025 Dec 3;7(6):fcaf474. doi: 10.1093/braincomms/fcaf474 (PMC12690201; doi:10.1093/braincomms/fcaf474)
Supplement: fcaf474_Supplementary_Data [file fcaf474_supplementary_data.docx]

# Supplementary material

## Models

$$\log\left( {RT}_{nm}-\exp\left( ndt \right) \right)\sim Normal\left( \mu_{nm},\sigma\right)$$

$$\mu_{nm}= \alpha+u_{subj\left[ n \right],1}+{\beta_{1}C}_{LFS\_vs\_HFS}+{\beta_{2}C}_{LFS\_vs\_OFF}$$

$ndt \sim$ *Uniform*(0, minimum reaction time)

$$\sigma\sim{Normal}_{+}(0, 0.5)$$

$$\alpha\sim{Normal}_{+}(6.5, 0.5)$$

$$\beta_{1} \sim Normal(0, 0.3)$$

$$\beta_{2} \sim Normal(0, 0.3)$$

$$u_{1} \sim Normal(0, 0.3)$$

Supplementary Equation 1 Shifted log-normal regression model and priors for the analysis of the response selection task reaction time data.

$${Error}_{nm} \sim Bernoulli\left( \theta_{nm} \right)$$

$$\eta_{nm}=\left( \frac{\exp(\theta_{nm})}{1+ \exp(\theta_{nm})} \right)$$

$$\eta_{nm}= \alpha+u_{subj\left[ n \right],1}+{\beta_{1}C}_{LFS\_vs\_HFS}+{\beta_{2}C}_{LFS\_vs\_OFF}$$

$$\alpha\sim Normal(-2, 1)$$

$$\beta_{1} \sim Normal(0, 1.5)$$

$$\beta_{2} \sim Normal(0, 1.5)$$

$$u_{1} \sim Normal(0, 1.5)$$

Supplementary Equation 2 Logistic regression model and priors for the analysis of the response selection task error data.

$$\log\left( {RT}_{nm}-\exp\left( ndt \right) \right)\sim Normal\left( \mu_{nm},\sigma\right)$$

$$\mu_{nm}= \alpha+u_{subj\left[ n \right],1}+{\beta_{1}C}_{Congruency}+{\beta_{2}C}_{LFS\_vs\_HFS}+{\beta_{3}C}_{LFS\_vs\_OFF}+{\beta_{4}C}_{LFS\_congruency\_vs\_HFS\_congruency}+{\beta_{5}C}_{LFS\_congruency\_vs\_OFF\_congruency}$$

$ndt \sim$ *Uniform*(0, minimum reaction time)

$$\sigma\sim{Normal}_{+}(0, 0.5)$$

$$\alpha\sim{Normal}_{+}(6.5, 0.5)$$

$$\beta_{1} \sim Normal(0, 0.3)$$

$$\beta_{2} \sim Normal(0, 0.3)$$

$$\beta_{3} \sim Normal(0, 0.3)$$

$$\beta_{4} \sim Normal(0, 0.3)$$

$$\beta_{5} \sim Normal(0, 0.3)$$

$$u_{1} \sim Normal(0, 0.3)$$

Supplementary Equation 3 Shifted log-normal regression model and priors for the analysis of the Flanker task reaction time data.

$${Error}_{nm} \sim Bernoulli\left( \theta_{nm} \right)$$

$$\eta_{nm}=\left( \frac{\exp(\theta_{nm})}{1+ \exp(\theta_{nm})} \right)$$

$$\eta_{nm}= \alpha+u_{subj\left[ n \right],1}+{\beta_{1}C}_{Congruency}+{\beta_{2}C}_{LFS\_vs\_HFS}+{\beta_{3}C}_{LFS\_vs\_OFF}+{\beta_{4}C}_{LFS\_Congruency\_vs\_HFS\_Congruency}+{\beta_{5}C}_{LFS\_Congruency\_vs\_OFF\_Congruency}$$

$$\alpha\sim Normal(-2, 1)$$

$$\beta_{1} \sim Normal(0, 1.5)$$

$$\beta_{2} \sim Normal(0, 1.5)$$

$$\beta_{3} \sim Normal(0, 1.5)$$

$$\beta_{4} \sim Normal(0, 1.5)$$

$$\beta_{5} \sim Normal(0, 1.5)$$

$$u_{1} \sim Normal(0, 1.5)$$

Supplementary Equation 4 Logistic regression model and priors for the analysis of the Flanker task error data.

$$\log\left( {RT}_{nm}-\exp\left( ndt \right) \right)\sim Normal\left( \mu_{nm},\sigma\right)$$

$$\mu_{nm}= \alpha+u_{subj\left[ n \right],1}+{\beta_{1}C}_{Go\_difference}+{\beta_{2}C}_{HFS}+{\beta_{3}C}_{OFF}+{\beta_{4}C}_{HFS*Go\_difference}+{\beta_{5}C}_{OFF*Go\_difference}$$

$ndt \sim$ *Uniform*(0, minimum reaction time)

$$\sigma\sim{Normal}_{+}(0, 0.5)$$

$$\alpha\sim{Normal}_{+}(6.5, 0.5)$$

$$\beta_{1} \sim Normal(0, 0.3)$$

$$\beta_{2} \sim Normal(0, 0.3)$$

$$\beta_{3} \sim Normal(0, 0.3)$$

$$\beta_{4} \sim Normal(0, 0.3)$$

$$\beta_{5} \sim Normal(0, 0.3)$$

$$u_{1} \sim Normal(0, 0.3)$$

Supplementary Equation 5 Shifted log-normal regression model and priors for the analysis of the Go-NoGo task reaction time data.

$${Error}_{nm} \sim Bernoulli\left( \theta_{nm} \right)$$

$$\eta_{nm}=\left( \frac{\exp(\theta_{nm})}{1+ \exp(\theta_{nm})} \right)$$

$$\eta_{nm}= \alpha+u_{subj\left[ n \right],1}+{\beta_{1}C}_{UncertainGo\_vs\_CertainGo}+{\beta_{2}C}_{UncertainGo\_vs\_NoGo}+{\beta_{3}C}_{LFS\_NoGo\_vs\_HFS\_NoGo}+{\beta_{4}C}_{LFS\_NoGo\_vs\_OFF_{NoGo}}{{+ \beta}_{5}C}_{LFS\_UncertainGo\_vs\_HFS\_UncertainGo}+{\beta_{6}C}_{LFS\_UncertainGo\_vs\_OFF\_UncertainGo}{{+ \beta}_{7}C}_{LFS\_CertainGo\_vs\_HFS\_CertainGo}+{\beta_{8}C}_{LFS\_CertainGo\_vs\_OFF\_CertainGo}$$

$$\alpha\sim Normal(-2, 1)$$

$$\beta_{1} \sim Normal(0, 1.5)$$

$$\beta_{2} \sim Normal(0, 1.5)$$

$$\beta_{3} \sim Normal(0, 1.5)$$

$$\beta_{4} \sim Normal(0, 1.5)$$

$$\beta_{5} \sim Normal(0, 1.5)$$

$$\beta_{6} \sim Normal(0, 1.5)$$

$$\beta_{7} \sim Normal(0, 1.5)$$

$$\beta_{8} \sim Normal(0, 1.5)$$

$$u_{1} \sim Normal(0, 1.5)$$

Supplementary Equation 6 Logistic regression model and priors for the analysis of the Go-NoGo task error data.

# Posterior Predictive Checks


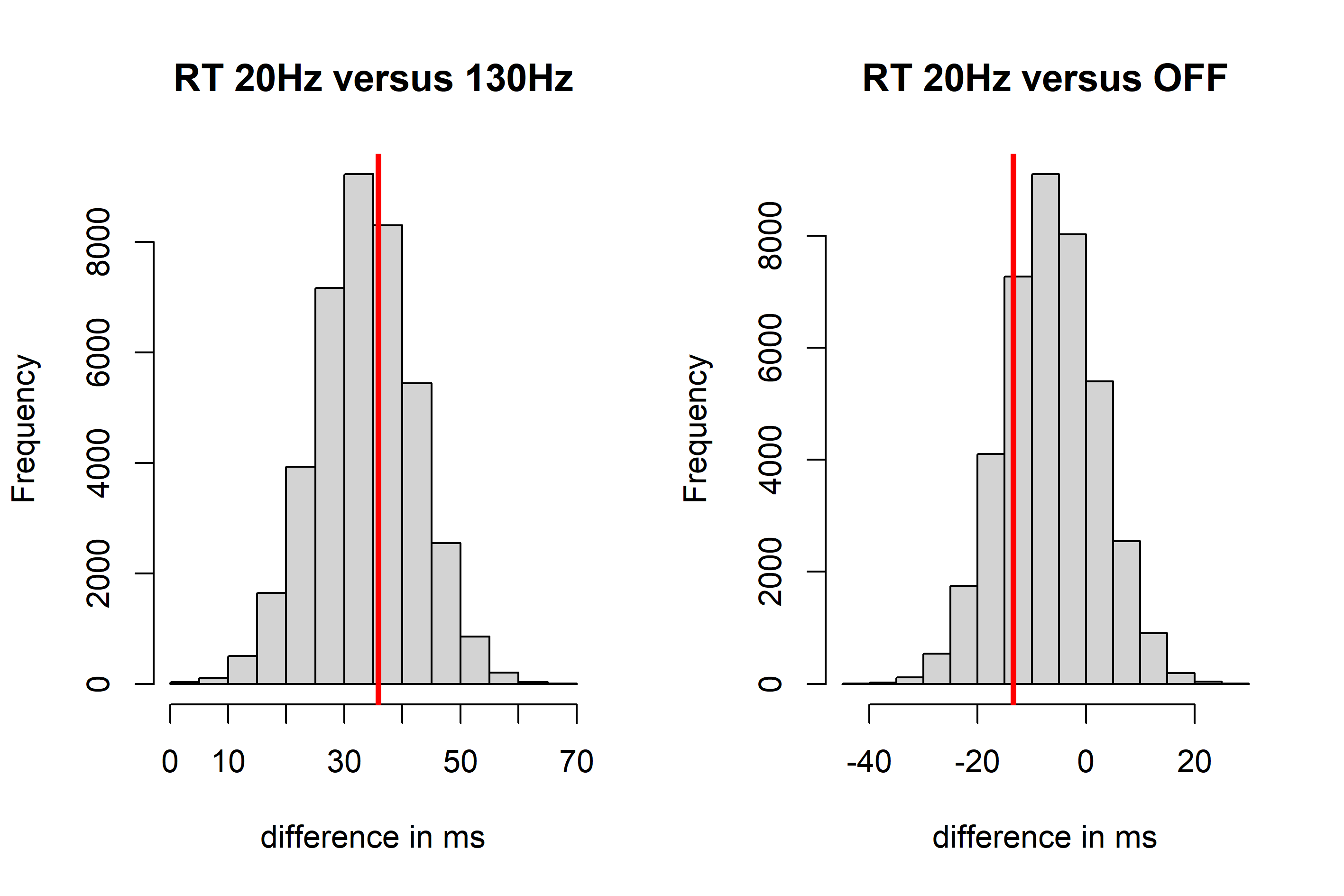


Supplementary Figure 1 Posterior predictive checks of reaction times for the analysis of the Response Selection task (N=17). The gray histogram depicts the posterior prediction for the reaction times, and the mean empirical reaction time is marked in red.


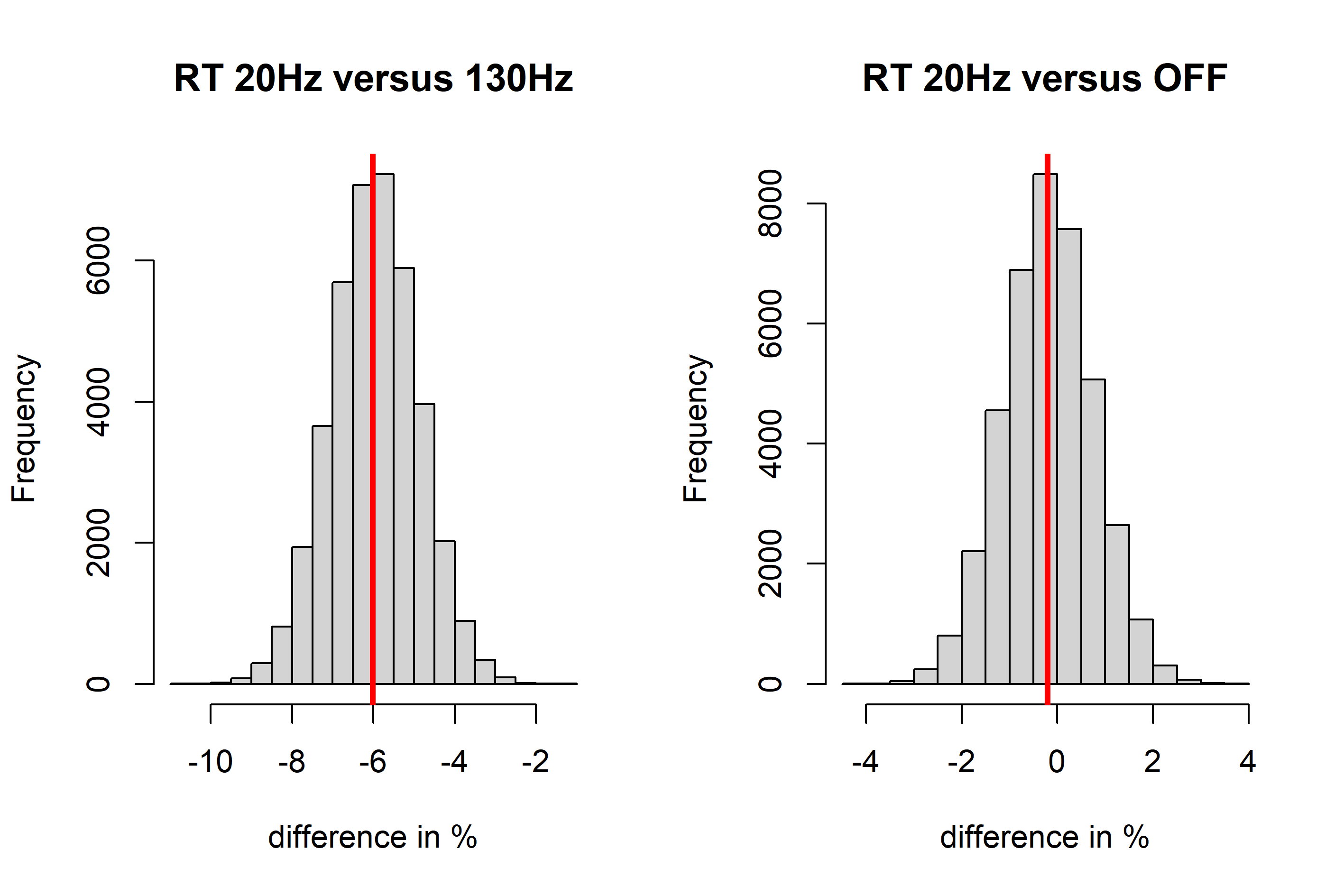


Supplementary Figure 2 Posterior predictive checks of error rates for the analysis of the Response Selection task (N=17). The gray histogram depicts the posterior prediction for the error rates, and the mean empirical reaction time is marked in red.


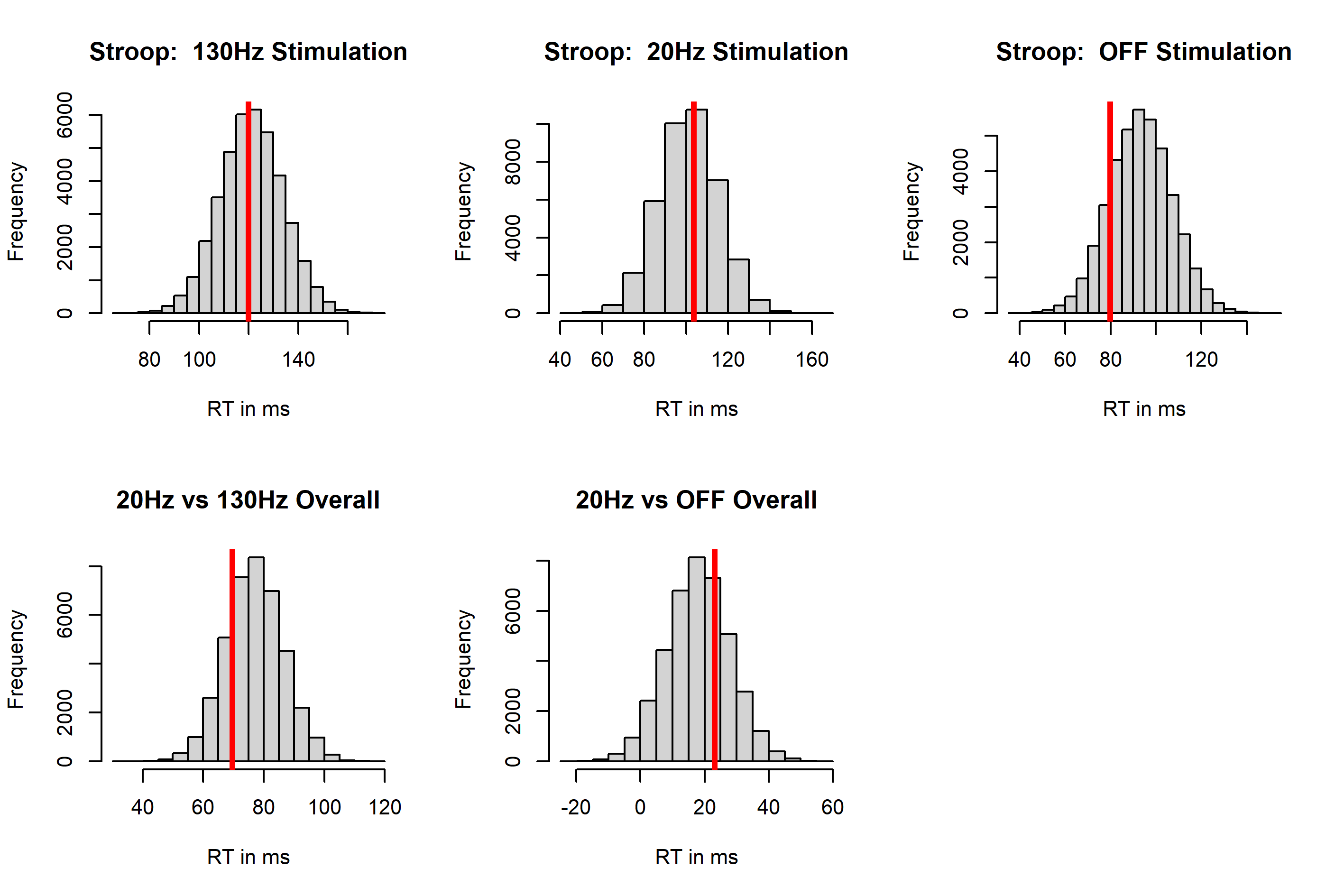


Supplementary Figure 3 Posterior predictive checks of reaction times for the analysis of the Flanker task (N=17). The gray histogram depicts the posterior prediction for the reaction times, and the mean empirical reaction time is marked in red.


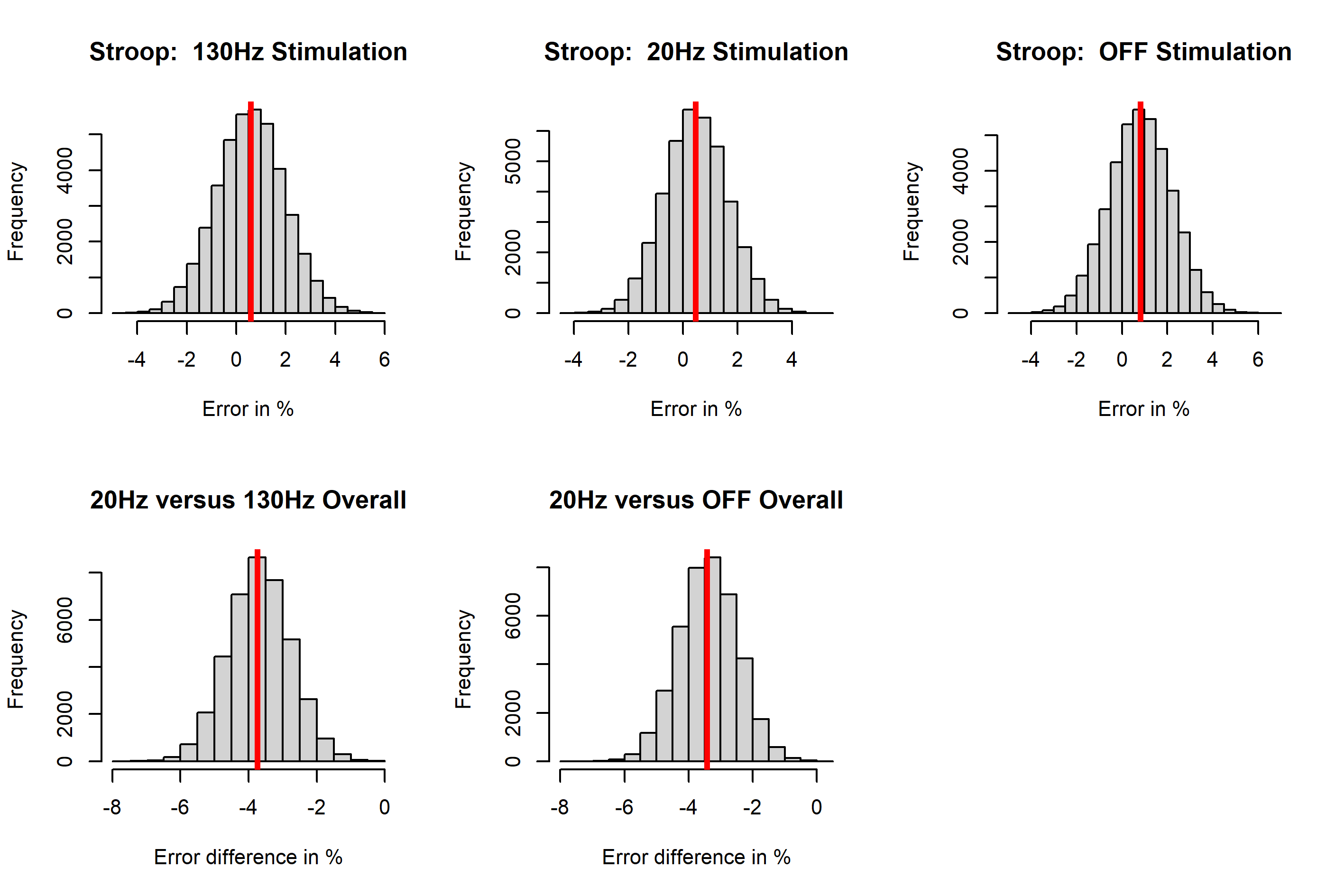


Supplementary Figure 4 Posterior predictive checks of error rates for the analysis of the Flanker task (N=17). The gray histogram depicts the posterior prediction for the error rates, and the mean empirical reaction time is marked in red.


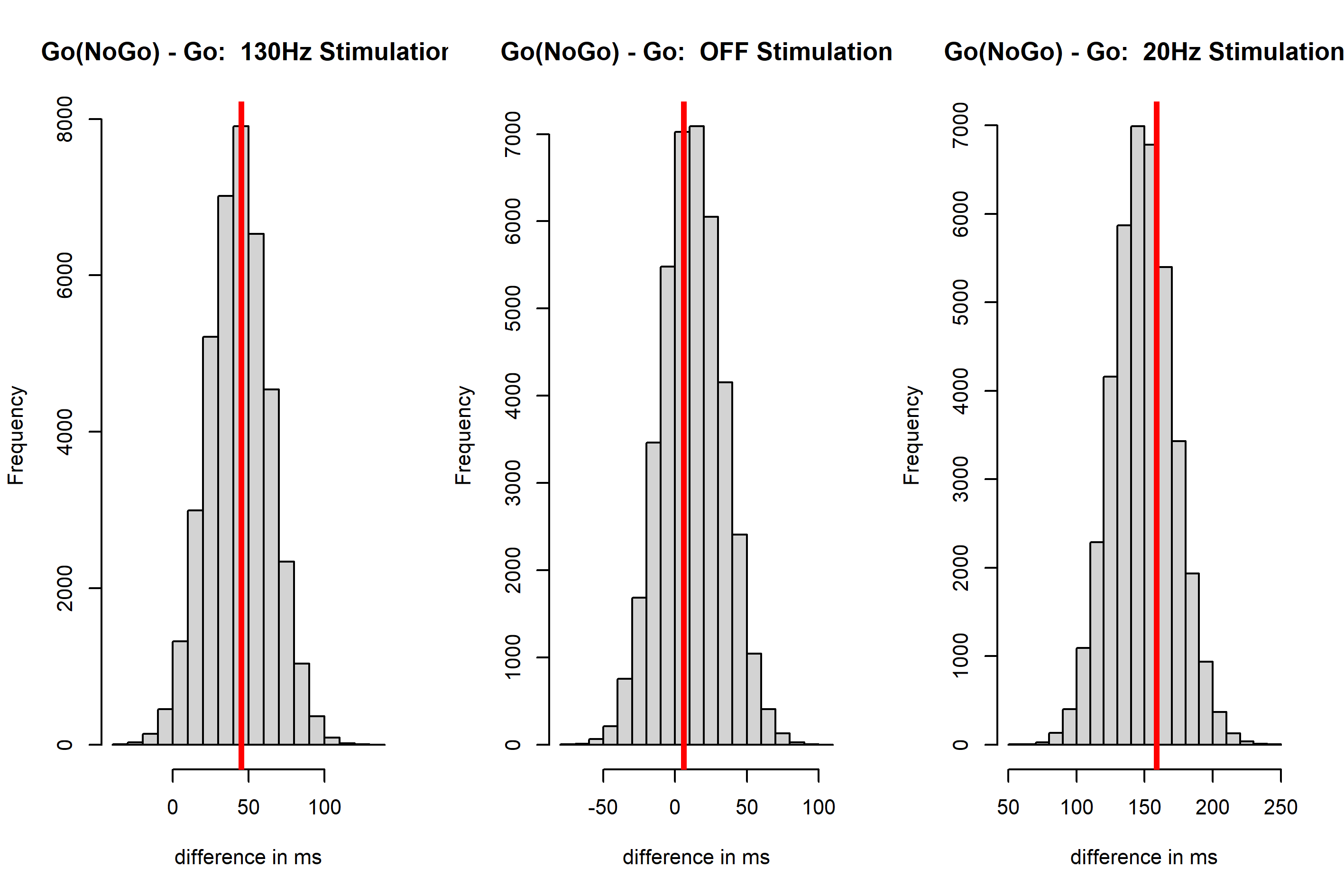


Supplementary Figure 5 Posterior predictive checks of reaction times for the analysis of the Go-NoGo task (N=17). The gray histogram depicts the posterior prediction for the reaction times, and the mean empirical reaction time is marked in red.


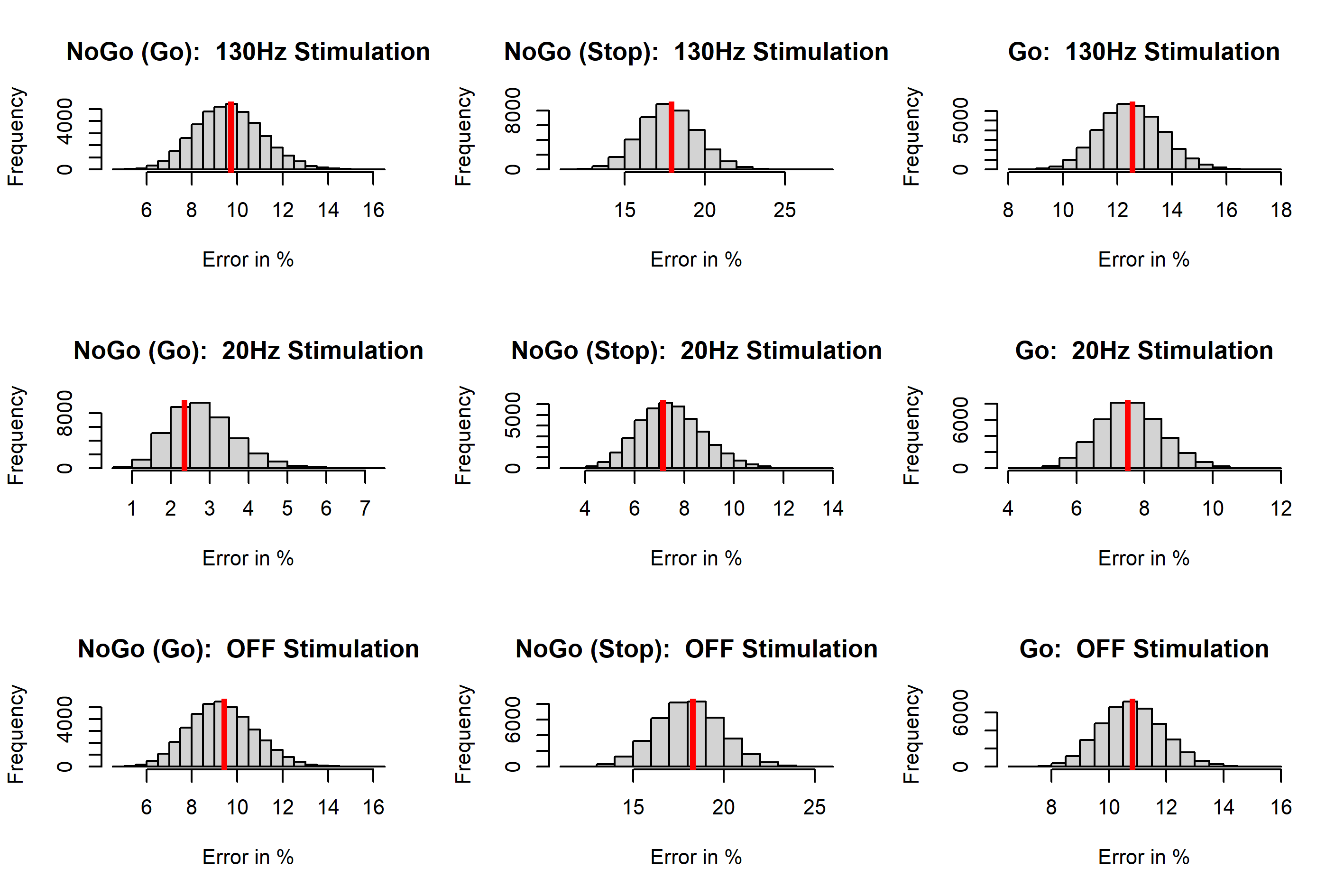


Supplementary Figure 6 Posterior predictive checks of error rates for the analysis of the Go-NoGo task (N=17). The gray histogram depicts the posterior prediction for the error rates, and the mean empirical reaction time is marked in red.


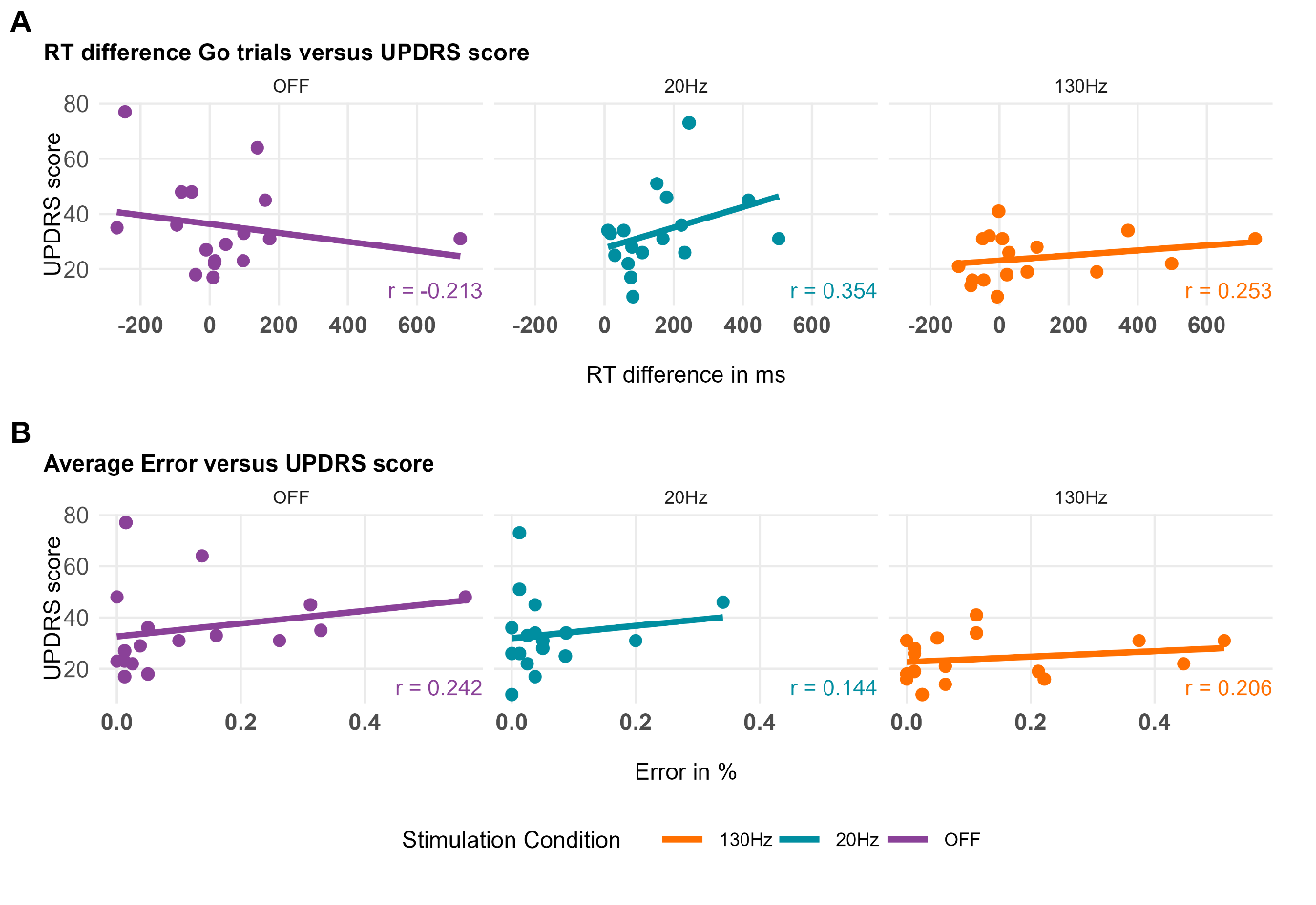


Supplementary Figure 7 Relationship between motor status and task performance across stimulation conditions (N=17). (A) Correlation between UPDRS-III scores and response slowing, measured as the reaction time difference between uncertain and certain Go trials. (B) Correlation between UPDRS-III scores and error rates. Pearson correlation coefficients (N=17) are shown for each stimulation condition. Solid lines represent linear regression fits.


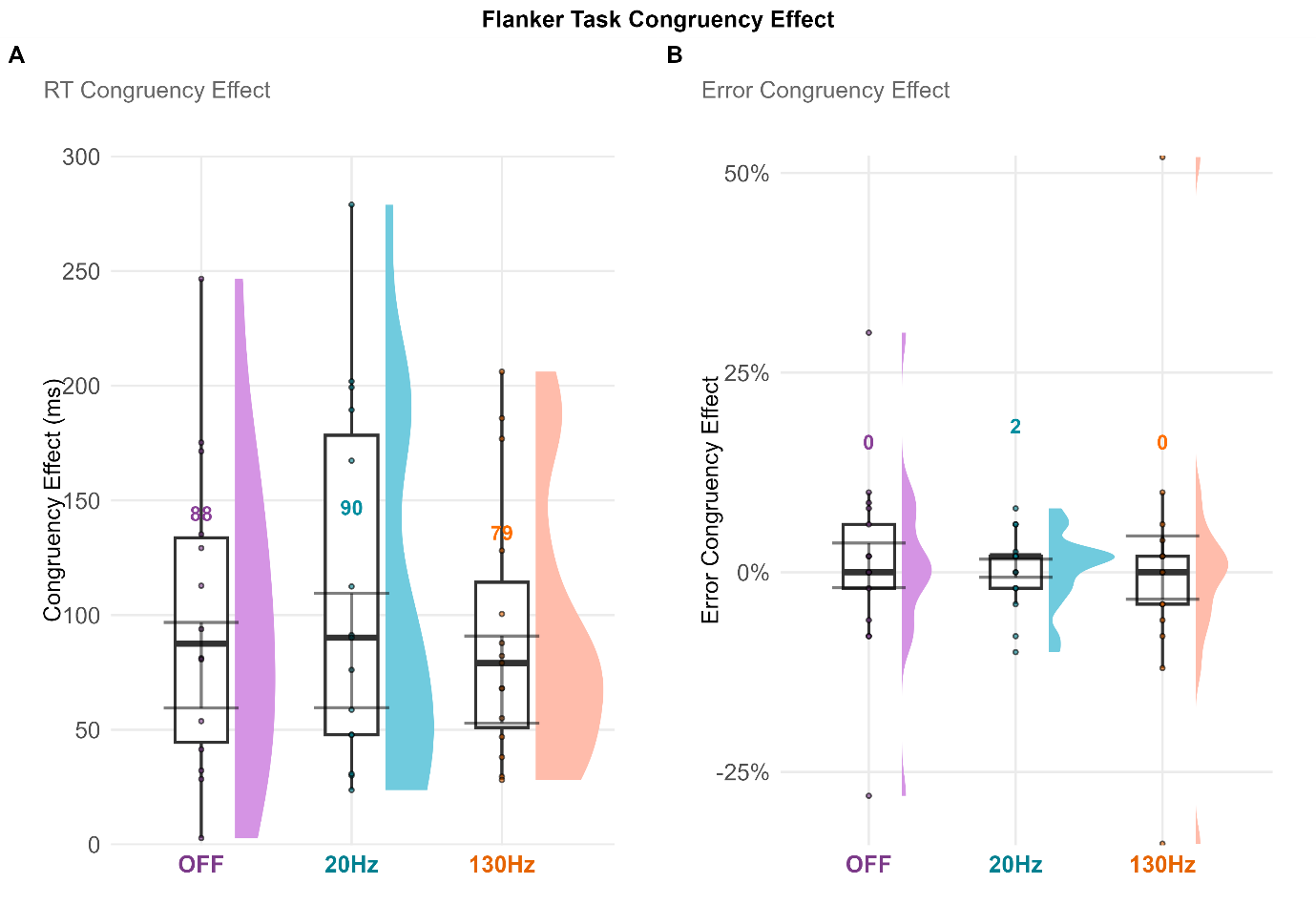


Supplementary Figure 8 Display of reaction time and error data by congruency condition (N=17) for the Flanker task (A, B). Numbers above the reaction time distributions (A) correspond to the median reaction time, and numbers above the bar graphs of the error data (B) correspond to the mean error rate for incongruent trials vs. congruent trials.
